# A nationwide inventory of the availability of alcohol-based handrub in Dutch acute care hospitals

**DOI:** 10.1186/1753-6561-5-S6-P261

**Published:** 2011-06-29

**Authors:** I Leroux-Roels, T van der Kooi, B van Benthem, V Erasmus, A Voss, T de Ruiter, G van Knippenberg-Gordebeke, MC Vos

**Affiliations:** 1 Medical Microbiology and Infectious Diseases, ERASMUS MC, Rotterdam, Netherlands; 2 RIVM, Bilthoven, Netherlands; 3 Department of Public Health, ERASMUS MC, Rotterdam, Netherlands; 4 Medical Microbiology, Radboud UMC, Nijmegen, Netherlands; 5 Infection Control, Groene Hart Hospital, Gouda, Netherlands; 6 IKNIP Consultancy , Venlo, Netherlands

## Introduction / objectives

Although there is general consensus that hand hygiene is the most effective measure to prevent healthcare associated infections, compliance is unacceptably low. Easy access to alcohol-based handrub at point of care (within 2m of the bed) is a main component of WHO’s multimodal strategy.

## Methods

We performed a descriptive study of alcohol-based handrub availability across 24 Dutch acute care hospitals to verify if difficult access could explain the national low compliance rate (19%). In each hospital the proportion of patient rooms with dispensers and the proportion of patient beds with point of care dispensers on an internal medicine, surgery, pediatrics and intensive care unit (ICU) ward was measured as well as the handrub consumption of the entire hospital. Hospitals reported the interventions they applied to increase compliance.

## Results

Handrub dispensers were present in 99.8, 100, 100 and 100% of patient rooms and at 50.8, 49, 57.1 and 83.8% of patient beds on the internal medicine, surgery, pediatrics and ICU wards, respectively. The average handrub consumption was 26.1 L/1000 nursing days (range: 8.6-51.7). No correlation was observed between handrub consumption and the percentage of beds with point of care dispenser. Of the 8 hospitals that reported doing interventions, 6 used written material and 7 used education, which is known to be not a very effective intervention.

## Conclusion

Enhanced access to alcohol-based handrub at point of care is required. In addition, the other components of the multimodal strategy need to be implemented to reach a sustained improvement of hand hygiene compliance.

## Disclosure of interest

None declared.

